# Supplementary material for: Deep-Channel uses deep neural networks to detect single-molecule events from patch-clamp data
Source: Commun Biol. 2020 Jan 7;3:3. doi: 10.1038/s42003-019-0729-3 (PMC6946689; doi:10.1038/s42003-019-0729-3)
Supplement: Supplementary file 2 — Supplementary Information [file 42003_2019_729_MOESM2_ESM.docx]

**Supplementary Information:**

**Supplementary Figures**

**Supplementary Figure 1 | Performance metrics for single and multiple ion channels during training and validation processes. a,** Validation accuracy for ion channel classification when only one channel is present; **ai,** by epoch over 50 epochs. **aii,** decrease in sparse categorical cross entropy loss function by epoch. **b,** As for **a** except now with several ion channel sojourns occurring simultaneously. **bi,** Increase in accuracy with epoch number over 50 epochs. **bii,** decrease in of sparse categorical cross entropy loss over 50 epochs.

**Supplementary Tables**

**Supplementary Table 1:** Performance evaluation for ion channel event classification using two different models: Baseline RNN without convolutional layers; and the proposed RCNN model

| **Metric** | **Baseline RNN** | | **RCNN (Deep-Channel)** | |
| --- | --- | --- | --- | --- |
|  | **Single-channel** | **Multi-channel** | **Single-channel** | **Multi-channel** |
| Accuracy | 0.913±0.026 | 0.876±0.012 | 0.992±0.004 | 0.988±0.003 |
| Precision | 0.918±0.024 | 0.882±0.014 | 0.994±0.003 | 0.991±0.002 |
| Recall | 0.908±0.018 | 0.869±0.011 | 0.991±0.005 | 0.986±0.004 |
| F-score | 0.913±0.025 | 0.875±0.14 | 0.992±0.004 | 0.988±0.003 |

**Supplementary Methods**

*Training performance parameters*

We first created simulated raw single ion channel recordings to determine if our RCNN network model could discriminate between open and close events in datasets when a single simulated ion channel was present (two-class classification; “open” v “closed”). We trained the RCNN classification model on simulated synthetic time series data from 3 distinct single ion channel datasets, with 10,000 records each.

A useful indication of training success is given by the accuracy (Acc), and specificity (Sp) metrics in performance evaluation to express the classification indicators. The equations to calculate Acc, and Sp are shown in equations s1 and s2, respectively.

| $Accuracy=\frac{TP+TN}{TP+FP+TN+FN}$ | (s1) |
| --- | --- |
| $Specificity=\frac{TN}{FP+TN}$ | (s2) |

With low numbers of channels present, after a few epochs of training, Deep-Channel gave near perfect classification accuracy with 99.2 ± 0.42 %, *n*=3 (Supplementary Figure 1A(i)). We then investigated the ability of the network to idealise/classify datasets with up to 5 ion channels present (6-class classification; closed or 1, 2, 3, 4 or 5 channels open). The average performance was achieved by applying our proposed RCNN model with only 2.64 ± 0.08 % (265 ± 84 out of 10000, *n=3*) wrongly classified events, as can be seen in Supplementary Figure 1B(i and ii), with 98.8 ± 0.45 % accuracy, and 2 ± 0.08 % loss.

We also compared the performance of our proposed RCNN model with a baseline RNN model without convolution layers, on both single and multiple ion channel event classification problems, the comparison established that the proposed RCNN model achieved better accuracy and loss (Supplementary Table 1).
