# Supplementary material for: Deep-Channel uses deep neural networks to detect single-molecule events from patch-clamp data
Source: Commun Biol. 2020 Jan 7;3:3. doi: 10.1038/s42003-019-0729-3 (PMC6946689; doi:10.1038/s42003-019-0729-3)
Supplement: Supplementary file 3 — Description of additional supplementary files [file 42003_2019_729_MOESM3_ESM.docx]

Description of additional supplementary items

Supplementary Data 1 is an excel file that shows the source data underlying Supplementary Figure 1.
